# Supplementary material for: Content-rich biological network constructed by mining PubMed abstracts
Source: BMC Bioinformatics. 2004 Oct 8;5:147. doi: 10.1186/1471-2105-5-147 (PMC528731; doi:10.1186/1471-2105-5-147)
Supplement: Additional File 2 — The original results of the above study (non-essential files are deleted to keep the file size under the limit set by BMC bioinformatics). [file 1471-2105-5-147-S2.bz2 › chilibotAdditionalFile2/dip20/281ID2498083E1104/html/FOS_JUNB.html]

 


 **FOS** and **JUNB** 
  
Found 830 abstracts in PubMed, retrieved 20.  
 

 What does Google say? 
 PDF only 
| .edu only 

---

**Interactive relationship** (e.g. stimulation, inhibition, etc)

**Neutral relationship**- In CL, c Jun, JunD,  **JunB**  and Fra 2 were present in DNA binding complexes and c  **Fos**  binding was not detected.  Ref: 12606371 Biol Reprod< MedlineTA>Biol Reprod,

**Non-interactive relationship** (e.g. studied together, co-existance, homology, etc.)

- Further EMSA analysis using antibodies to various AP 1 components revealed that  **junB**  antibodies partially depleted the increase in binding to the PRE1 seen in UK rap1 cells while antibodies to other AP 1 constituents such as c jun, c  **fos** , and ATF 1 had no effect on binding.  Ref: 12447993 J Cell Physiol, 2003
- Phosphorylated c Jun,  **JunB** , JunD, and Fra 1, but not c  **Fos** , FosB, or ATF 2, are detected in the AP 1 DNA binding complex in cells exposed to trivalent arsenicals.  Ref: 12475910 FASEB J, 2003
- Supershift electrophoretic mobility shift assay indicated that  **JunB** , JunD, and c  **Fos**  components of AP 1 were particularly affected.  Ref: 12857751 J Biol ChemJ Biol Chem, 2003
- Immunoblot analyses confirmed that c Jun, JunD,  **JunB** , c  **Fos** , Fra 1, Fra 2 and FosB immunoreactive proteins were present in whole cell extracts of all antral follicles and midluteal phase corpora lutea as well as granulosa cells isolated from different sized antral follicles.  Ref: 12606371 Biol Reprod< MedlineTA>Biol Reprod, 2003
- Levels were highest shortly after transient DC shifts, and decreased thereafter mono exponentially with half lives of 4 5 and 58 min for c  **fos** ,  **junB**  and MKP 1 mRNAs, respectively.  Ref: 12671253 Restor Neurol Neurosci< MedlineTA>Restor Neurol Neurosci, 2003
- Nuclear extracts from granulosa cells from 1 2 or 8 10 mm antral follicles bound an AP 1 DNA consensus sequence and complexes consisted predominantly of c Jun, JunD,  **JunB** , c  **Fos** , and Fra 2.  Ref: 12606371 Biol Reprod< MedlineTA>Biol Reprod, 2003
- In a Gal4 fusion assay, Pdcd4 specifically inhibited activation of c Jun and c  **Fos**  activation domains, but did not inhibit activation of  **JunB** , JunD, Fra 1, or Fra 2.  Ref: 12802278 Oncogene, 2003
- In contrast, mice exposed and re exposed only to the context, without foot shock, did not show behavioral signs of contextual fear conditioning and exhibited significantly less expression of c  **Fos**  and  **JunB**  in CA1 neurons.  Ref: 12882314 Genes Brain Behav, 2003
- In these mice context dependent memory retrieval evoked a marked induction of c  **Fos**  and  **JunB** , but not of c Jun and JunD, in pyramidal CA1 neurons of the dorsal hippocampus.  Ref: 12882314 Genes Brain Behav, 2003
- Genes that were commonly down regulated in both PB T cells and Jurkat T cells included CDK inhibitors p16, p19, p27, proapoptotic caspases, and the transcription factors c  **fos**  and jun B  [ **JUNB** ] .  Ref: 12808112 Lab Invest, 2003
- Towards this end, we characterized the effect pharmacological disruption of the MAPK cascade has on the expression of the immediate early genes c  **Fos** ,  **JunB**  and EGR 1.  Ref: 12752379 Eur J Neurosci, 2003
- Mechanistically, sodium valproate and pyridoxine significantly attenuated domoic acid induced increase in levels of glutamate, increase in levels of calcium influx, decrease in levels of gamma aminobutyric acid and increase in levels of the protooncogenes c  **fos** , jun B  [ **JUNB** ]  and jun D.  Ref: 12686137 Biochim Biophys Acta, 2003
- Memory retrieval after contextual fear conditioning induces c  **Fos**  and  **JunB**  expression in CA1 hippocampus.  Ref: 12882314 Genes Brain Behav, 2003
- Role of CREB transcription factor in c  **fos**  activation in natural killer cells.In natural killer cells, interleukin 2 differentially regulates the expression of several transcription factors, including  **JunB**  and c  **fos** .  Ref: 12432566 Eur J Immunol, 2002
- In injured animals without SD, only a short lasting response of c  **fos** ,  **junB** , c jun and MKP 1 mRNAs as well as c  **Fos**  protein was bilaterally found in the piri form cortex, and with ipsilateral dominance the dentate gyrus and hippocampal CA3 4 fields at 1 h after lesioning.  Ref: 12671253 Restor Neurol Neurosci< MedlineTA>Restor Neurol Neurosci, 2002
- In situ hybridization using oligonucleotide probes was used to study the effects of intrastriatal microinjection of corticoliberin on the expression of the early genes c  **fos** , jun B  [ **JUNB** ] , c jun, and NGFIA in the rat brain.  Ref: 12617307 Neurosci Behav Physiol, 2003
- Administration of corticoliberin into the neostriatum induced the expression of mRNA encoded by the early genes c  **fos** , jun B  [ **JUNB** ] , and NGFIA in both the neostriatum itself and in its efferent structures, particularly the nucleus accumbens and various parts of the cortex.  Ref: 12617307 Neurosci Behav Physiol, 2003
- Among the early response genes analyzed, c myc,  **junB** , junD, c jun, c  **fos** , fosB, fra, as well as max, mad1 4, sin3, only c jun and fra 2 mRNAs were up regulated after 5D exposure.  Ref: 12648525 J Steroid Biochem Mol Biol, 2003
- Acute injection of morphine induces expression of the immediate early genes c  **Fos**  and  **JunB**  in several forebrain regions of the rat, in part through an N methyl D aspartate NMDA receptor dependent mechanism.  Ref: 12809699 Neuroscience< MedlineTA>Neuroscience, 2003
- Using specific polyclonal antisera against c  **Fos** ,  **JunB** , c Jun and JunD, we tried to identify the candidate transcription factors of the immediate early gene family which may contribute to the molecular processes during contextual memory reconsolidation.  Ref: 12882314 Genes Brain Behav, 2003
- The expression of c  **fos** ,  **junB**  and MKP 1 mRNAs was closely related to the time interval between the last spreading depression and the end of the experiments.  Ref: 12671253 Restor Neurol Neurosci< MedlineTA>Restor Neurol Neurosci, 2003
- On the other hand, expressions of proto oncogene c jun,  **junB**  and c  **fos**  were induced by TPA and Saikosaponin a during 30 min to 6 h of treatment.  Ref: 12592382 Oncogene, 2003
